# Improving preconception health and care: a situation analysis

**DOI:** 10.1186/s12913-017-2544-1

**Published:** 2017-08-23

**Authors:** Ashley Goodfellow, John Frank, John McAteer, Jean Rankin

**Affiliations:** 1 Department of Public Health, NHS Lanarkshire, Bothwell, G71 8BB, Glasgow, Scotland; 2 Scottish Collaboration of Public Health Research and Policy, Centre for Population Health Sciences, University of Edinburgh, EH48 9DX Edinburgh, Scotland; 3 Maternal Child and Family Health, School of Health Nursing and Midwifery, University of the West of Scotland, Paisley, PA1 2BE Scotland

**Keywords:** Preconception health, Preconception care, Interconception, Pregnancy and birth, Prevention, Preventative spend, Childbearing age, Qualitative research

## Abstract

**Background:**

The purpose of this situation analysis was to explore the views of health and non-health professionals working with women of childbearing age on current and future delivery of preconception care in one National Health Service (NHS) Board area in Scotland.

**Methods:**

The situation analysis was undertaken using a mixed methods approach. Six focus groups were conducted organised by profession – general practitioners (GPs), practice nurses, health visitors, family nurses, guidance teachers and youth workers. Existing evidence of effective preconception care interventions informed focus group guides. A survey was undertaken with community pharmacists which provided qualitative data for analysis. Focus group transcripts were analysed by two researchers using a thematic analysis approach.

**Results:**

There was lack of awareness of preconception health and its importance amongst the target group. Levels of unplanned pregnancy hampered efforts to deliver interventions. Professional knowledge, capacity and consistency of practice were viewed as challenges, as was individual compliance with preconception care advice. Improvement requires multifaceted action, including ensuring the school curriculum adequately prepares adolescents for future parenthood, increasing awareness through communication and marketing, supporting professional knowledge and practice and capitalising on existing opportunities for preconception care, and ensuring services are equitable and targeted to need.

**Conclusions:**

Delivery of preconception care needs to be improved both before and between pregnancies to improve outcomes for women and infants. Action is required at individual, organisational and community levels to ensure this important issue is at the forefront of preventative care and preventative spending.

## Background

The early years of a child’s life are critical in shaping future health and wider outcomes and it is acknowledged that maternal health before and during pregnancy impacts on the health of the child long after infancy [1]. There are many interventions that can improve maternal and child health outcomes if provided before or early in pregnancy. This preconception care is defined as ‘a set of interventions that aim to identify and modify biomedical, behavioural and social risks to a woman’s health or pregnancy outcome through prevention and management, emphasizing those factors that must be acted upon before conception or early in pregnancy to have maximal impact’ [1]. The negative social consequences and major financial costs of poor pregnancy and birth outcomes has been highlighted [2]. Preconception care is a key area for preventative spending.

Poor maternal lifestyle and health prior to and during pregnancy increases risk of a range of poor pregnancy and birth outcomes. In Scotland, the majority of women report taking folic acid supplements in the first trimester of pregnancy but less than half (40%) report consumption prior to conception [3]. Rates of antenatal diagnosis of neural tube defects (NTDs) are 1.02 per 1000 births, including terminations and stillbirths [4]. Almost one fifth of women (18.1%) are obese during pregnancy which may contribute to rising levels of induction (25.4/1000 live births) and caesarean sections (15.7/1000 live births emergency and 12.8/1000 live births elective) [5]. Around one fifth (18.4%) of women in Scotland smoke during pregnancy which increases the risk of premature birth and intra-uterine growth restriction (IUGR) [5]. Around 59% of premature births in Scotland are low or very low birth weight [5]. Survey data highlight that 87% of women reported consuming alcohol before pregnancy and 35% during pregnancy [3]. Alcohol consumption in pregnancy increases risk of miscarriage, premature birth, low birth weight and fetal alcohol spectrum disorder (FASD). Due to the challenges associated with diagnosis, rates of FASD in Scotland are not available although estimates would suggest that 9 in every 1000 births may be affected [6]. Drug misuse affects 5.9 per 1000 live births in Scotland [5]. No data were available on levels of gender-based violence before or during pregnancy or on the uptake of Rubella, Varicella and Hepatitis B vaccines in women of childbearing age and those in high risk or eligible groups. Many of the aforementioned risk factors and outcomes are worsened with increasing deprivation.

A range of preconception care interventions are available, some with more robust evidence of effectiveness than others. Folic acid supplementation, weight management and smoking cessation interventions were shown to be effective in the preconception period, if delivered at the appropriate intensity and duration [7–13]. Evidence relating to interventions to reduce or abstain from alcohol consumption prior to or during pregnancy is limited [6, 14]. There is good evidence of interventions to reduce drug misuse but a lack of evidence in relation to the benefits of screening asymptomatic individuals in the preconception period [15]. Limited evidence shows that there is benefit in screening for gender-based violence and intervening prior to conception [16]. The efficacy of vaccines recommended in the preconception period is undisputed [17] yet evidence in relation to how best to ensure uptake in eligible women is lacking. There is also a lack of evidence on screening and interventions for mental health in the preconception period, other than for women with a pre-existing mental health condition [18].

National policy and legislation supports a shift to prevention and early intervention, which includes reviewing the way in which maternity and health visiting services are delivered. This provided a foundation on which to undertake the situation analysis on which this paper reports.

## Methods

### Aim

To explore the views of health and non-health professionals working with women of childbearing age on current and future delivery of preconception care in one NHS Board area in Scotland.

### Study design

A mixed methods approach to data collection was applied to obtain the views of stakeholders. Qualitative focus group research was used to gather the views of a range of professionals working with women of childbearing age: general practitioners (*n* = 7), practice nurses (*n* = 2), health visitors (*n* = 4), family nurses (*n* = 3), secondary school guidance teachers (*n* = 3) and youth workers (*n* = 8). Focus groups were organised by type of professional. In addition, an online questionnaire survey was undertaken with community pharmacists.

### Setting

The situation analysis was conducted within NHS Lanarkshire. In 2014, Lanarkshire had a population of 653,310 comprising two coterminous local authority areas – North Lanarkshire and South Lanarkshire [19]. Lanarkshire is the third largest health board area in Scotland next to Greater Glasgow and Clyde (population 1,142,580) and Lothian (population 858,090). The birth rate in 2014 was 56.7/1000 women (54.8/1000 in Scotland). Lanarkshire is made up of 726 datazones (small area geographies comprising 500–1000 residents), 132 of which were in the 15% most deprived datazones in Scotland (18.2% of the local population). This is only surpassed by Greater Glasgow and Clyde (45.4%) and Ayrshire and Arran (19.8%) [20]. Lanarkshire has a marginally higher maternal obesity rate than Scotland (19.7% compared to 18.1%) and has slightly higher rates of induction (27.3/1000 live births) and caesarean sections (16.5/1000 live births emergency and 13.9/1000 live births elective) [5]. Smoking in pregnancy is comparable with the national rate (18.5% compared to 18.4%), with similar rates of premature birth (7% of total births) and low birth weight (6% of live births) [5]. Drug misuse affects 4.8/1000 live births in Lanarkshire [5].

### Recruitment

Purposeful sampling was employed to recruit individuals from specific professional groups. Contact was made through the appropriate NHS or Local Authority senior manager and information about the research was provided. A short PowerPoint presentation summarising the evidence base on effective preconception care interventions was also provided to participants. Potential participants were invited to review the information and volunteer to take part in a focus group if they so wished. The sample size was not based on criteria for thematic saturation. The online survey was distributed to all community pharmacists in Lanarkshire (*n* = 143) and responses invited within 4 weeks. A covering letter accompanied the survey which explained the purpose of the research and encouraged pharmacists to share their views.

### Data collection and management

Two focus group guides were developed to elicit the views of participants; one for health professionals and one for non-health professionals. Focus group guides were only marginally different to suit the needs of the profession and workplace setting. Topics covered included: awareness of the importance of preconception health, current delivery of preconception care interventions, how effectively interventions were delivered, and challenges, solutions and areas for future action (see Appendix). Focus groups were digitally recorded with permission and transcribed verbatim. Online survey questions were derived from the focus group guide and developed using the Survey Monkey web-based resource.

### Data analysis

Transcripts were analysed by two of four authors using thematic analysis [21]. Transcripts were firstly read thoroughly to gain a sense of the whole content of discussions and so that the researchers could become familiar with the data. For each transcript, significant statements were identified and highlighted; these were then extracted for each professional group and meanings formulated. These formulated meanings were then further categorised into theme clusters and emergent themes, under which the study findings can be described. Disagreements in thematic categories were minimal and resolved through discussion between the two researchers until consensus was reached.

## Results

Twenty-seven professionals participated in the focus group research. This included 7 GPs and 2 practice nurses from a total of 105 practices, 4 of 16 health visitor team leaders, and 3 of 16 family nurses. Three guidance teachers from a total of 23 secondary schools and 8 of approximately 40 youth workers also participated. Community pharmacists were included in the research as they are an important group of health professionals who have the opportunity to interact with large segments of the population and promote and support preconception health and care. Twenty-eight community pharmacists responded to the survey (20% response rate) which was deemed too small to present quantitative results, however, free text survey responses were included in the thematic analysis.

**Fig. 1:**
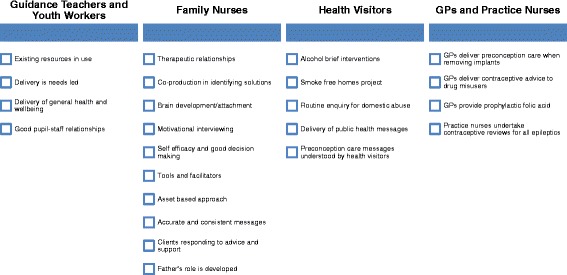
Identified good practice

**Fig. 2:**
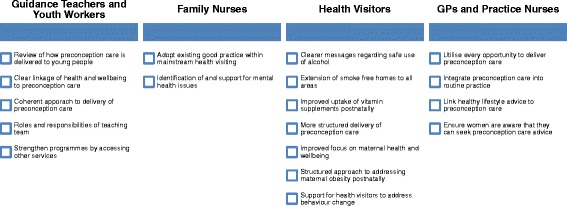
Areas for improvement

**Fig. 3:**
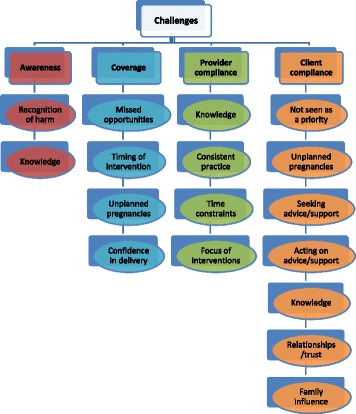
Preconception care challenges

**Fig. 4:**
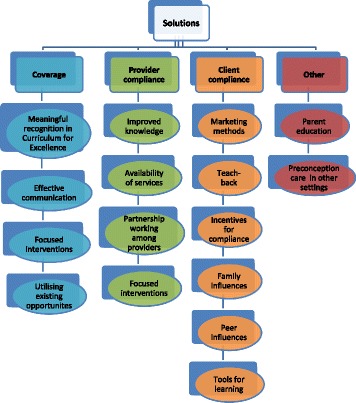
Solutions for improved preconception care

Three main areas for exploration were identified. These were: current good practice and areas for improvement, challenges relating to delivery of preconception care, and solutions to improve preconception care. Given the small number of focus group participants, data on current good practice and areas for improvement (Figs. 1 and 2) were appropriately aggregated for some professional groups. General practice staff (GPs and practice nurses) were combined. Non-health staff (school guidance teachers and youth workers) were also combined because they work with the same target group (young people aged 12–18 years). Although working with similar client groups, data from health visitors and family nurses were reported separately as the specific programmes they deliver may have influenced discussions and responses. Data from all professional groups (including community pharmacists) were used to present results on the challenges and solutions to delivering preconception care (Figs. 3 and 4).

### Current practice

Figures 1 and 2 set out current good practice and areas for improvement categorised across professional groupings. Secondary school guidance teachers and youth workers had the most opportunity to work with young people prior to conception. Good pupil-staff relationships existed and youth work was needs-led. General health and wellbeing was delivered across the curriculum, however this occurred in a fragmented way and not linked to a particular life stage:

*“We tend to have input from several departments so it’s not focused on one area so PE might do sort of general exercise and fitness and home economics will do just healthy eating”* [Guidance Teacher].

Good practice was identified across the Family Nurse Partnership (FNP) programme; which is an evidence-based prescriptive early intervention programme to support young, first time mothers from pregnancy to 2 years postnatal. The programme supports development of family nurse-client therapeutic relationships which promotes trust, co-production and self-efficacy. Identification and management of adolescent mental health problems was identified as an area requiring further support, particularly in those who were disengaged from education and therefore support services.

Health visitors delivered a universal pathway of care and support to children and families, including key public health messages in the interconception period. The revised (2015) pathway increases the number of contacts with families and was felt to be an opportunity to provide focused input on maternal health and a structured approach to preconception care. A consistent message about safe alcohol use before and during pregnancy was lacking.

GPs rarely had contact with women specifically about preconception care. Addressing fertility concerns or providing contraceptive advice was common. Prophylactic folic acid was prescribed for women unsure of whether to continue their pregnancy. Practice nurses had a range of opportunities where it was appropriate to engage with women about preconception care, including contraceptive reviews and removal of contraceptive implants, which could be capitalised on more fully. It was felt that women may be unaware that they can seek advice and support for preconception care:

*“If this is going to come up at different appointments, it’s the time it takes as well, you know… that should be an appointment on its own…and I don’t know if women would know that they could do such a thing as make an appointment for preconception care”* [Practice Nurse].

### Challenges

Figure 3 sets out the challenges associated with providing preconception care relating to awareness, coverage (delivering preconception care to the right people at the right time), provider compliance (providing accurate and consistent information), and individual compliance (individuals adhering to or acting upon advice and interventions).

Professional groups felt there was a lack of awareness in women of childbearing age (and their significant others) about preconception care and its importance to maternal and child health. This included obesity not being recognised as an issue and normalisation of gender-based violence. There was limited awareness of the role of health services in preventative care.

The major challenge in terms of coverage was the level of unplanned pregnancy. Women were not actively planning pregnancy and therefore taking steps to improve their health prior to conception. The view was that generally healthy women would not access their GP and even those actively planning pregnancy tended not to seek advice from their GP or practice nurse. Even during contacts, women did not necessarily disclose that they were planning pregnancy:

*“People don’t talk to us and say they’re going to get pregnant”* [Practice Nurse].

Four key challenges emerged relating to provider compliance; knowledge, consistency of practice, time constraints and focus of interventions. Lack of knowledge of preconception care messages was identified across some professional groups and about specific topics such as weight management. Inconsistent information and lack of uniform practice were also highlighted. The focus of interventions was identified as a challenge, specifically by guidance teachers and youth workers, whose focus was on safe sex and prevention of pregnancy rather than preconception care. This led some to believe that the curriculum was not fit for purpose as young people were not being equipped with appropriate life skills for future parenthood:

*“We spend that much time to get them to avoid having sex, you know… we’re not really preparing them for … future life, we’re trying to think about here and now”* [Guidance Teacher].

A range of challenges were identified in relation to individual compliance. There was a general view that women did not seek preconception care either because of a lack of knowledge and/or planning. Professionals felt that preconception care was not viewed as a priority for women of childbearing age. Not taking vitamin supplements when provided free of charge was given as an example. Women may not understand the impact of their health on that of their child, as healthy babies can be born to mothers who have a poor lifestyle. Acting on advice given and behaviour change is difficult for some women, particularly in terms of addressing addictions or losing weight. Trusting professional-client relationships was seen as vital; however, time to develop such relationships was limited.

### Solutions

Figure 4 outlines the solutions identified by professionals to improve preconception care relating to coverage, provider compliance, individual compliance and other solutions. The school curriculum was viewed as a key mechanism to support coverage. Preconception care should be meaningfully built into a comprehensive curriculum on healthy relationships, sexual health and preparation for parenthood, delivered by well-informed and confident staff. Effective communication to promote and raise awareness of preconception care was suggested, including more effective use of social media and mobile phone apps.

Focused interventions, targeted at those most in need, were deemed important in terms of reducing inequalities:

*“…you are more likely to, because you are disengaged from education, school or unemployment, you’re more likely to smoke heavy, drink heavy, be obese”* [Youth Worker].

During the interconception period, focused, person-centred intervention at around 12 months postnatal was felt appropriate as part of the revised universal health visiting pathway. Existing opportunities for preconception care should be capitalised upon, including, sexual health and family planning services, dedicated youth health services, contraceptive reviews and health reviews for postnatal mothers.

Provider compliance improvements included enhancing knowledge, availability of services, partnership working and the focus of interventions. Non-health professionals felt that the NHS should provide them with accurate and consistent preconception care information and supporting materials. Community pharmacists and practice nurses in particular identified training needs. A consistent message on alcohol use was needed, as well as equitable access to services across urban and rural areas and uniformity of roles/consistency of practice.

Solutions were identified to encourage individual compliance with preconception care advice. Marketing methods should be employed to illustrate the importance of maternal health before and during pregnancy, although there were conflicting views about the use of shock-tactics or more subtle messaging. The importance of teach-back was highlighted by nursing staff to reinforce advice and assess understanding. Capitalising on incentives to comply with preconception care advice was identified as a solution; none more so than building on the motivation future parents have to do the best for their child. Family and peer influences could be used in a positive way to influence behaviour, for example peer education videos.

Other solutions identified included parent education and relevance of preconception care in other settings, such as further education establishments and workplaces. Parent education was viewed as important as many feel uncomfortable discussing sensitive issues with their children. Parents need to feel confident about providing accurate advice as the home environment is where young people are likely to seek guidance and spend a lot of time.

## Discussion

There is widespread understanding that the health of the mother affects the health of the child long after infancy [1]. “Fetal programming” means that certain risk factors during pregnancy e.g. smoking, obesity, alcohol consumption, can change the expression of certain genes during development resulting in longer term effects on child and adult health [22]. The fetal programming hypothesis and local statistics reinforce the need for preconception care to optimise the mother’s health before she conceives. Evidence shows that preconception care interventions can be effective if delivered at the appropriate intensity and duration [8]. These interventions are important as prevention and early intervention is essential for improving pregnancy and birth outcomes for women of childbearing age [1, 2].

The situation analysis identified good practice in the delivery of preconception care interventions although practice was not consistent across and between professional groups. The FNP programme in particular had many identified strengths. Improvements were identified across all professions. Challenges in improving preconception care included: a lack of awareness of what preconception care is and why it is important; levels of unplanned pregnancy; professional knowledge, capacity and consistency of practice; and individuals seeking and acting upon preconception care advice.

Improving preconception care requires action across professions and settings. In terms of coverage, building on the Curriculum for Excellence, better communication and focussed interventions with the target group, and capitalising on existing opportunities for preconception care were all identified as areas for improvement. Enhanced training and awareness sessions for professionals to improve knowledge and consistency of practice was essential, as was ensuring equitable service provision and targeting services to need. A number of solutions were identified to improve individual compliance with interventions, mostly centred around increasing awareness through a range of marketing methods and using learning tools, teaching methods and ‘incentives’ which have demonstrated positive impact. Support for parents and the wider family should be provided so that they are enabled to support future generations in making informed decisions about health, relationships and parenting.

The situation analysis has yielded interesting results at NHS Board level but has a number of limitations. Firstly, the evidence base relating to effective preconception care interventions is small and highlights the challenges associated with conducting research in this area. Collation of data was challenging and was not available at national or local level for some pregnancy and birth risk factors and outcomes. The lack of data on FASD demonstrates the complexity associated with identification and diagnosis and therefore, its potential impact on future service provision. Finally, the number of participants in some focus groups was small. Recruitment for focus groups during busy periods was difficult and this will affect the representativeness and generalizability of results.

## Conclusions

This study sought to understand the views of both health and non-health professionals working with women of childbearing age on current and future delivery of preconception care in one NHS Board area in Scotland. NHS Boards cover a specific geographical area in Scotland and are responsible for protecting and improving population health and delivering healthcare services. The study has allowed a review of local practice against preconception care interventions known to be effective. The review has focused on the challenges and solutions to delivering effective preconception care to women of childbearing age and has made a number of recommendations for improvement, set within the local context. These are:

- The school curriculum requires review in line with the national Pregnancy and Parenthood in Young People Strategy to ensure it is fit for purpose in order to meet young people’s needs.
- The NHS and partner agencies need to increase awareness of preconception care across all appropriate settings using a range of communication and marketing methods. All existing opportunities for preconception care across professions and settings should be explored, including, targeted preconception care interventions for vulnerable groups of young people.
- Training on preconception care is required for both health and non-health professionals.
- The universal pathway for health visiting needs to specifically include delivery of preconception care at appropriate intervals postnatally and the principles, tools and facilitators employed in FNP need to be adopted for use across health visiting practice.
- The NHS and partner agencies need to explore how best to deliver evidence-based interventions to support weight management and mental wellbeing in the preconception and interconception periods and to prevent and diagnose risk of fetal alcohol spectrum disorder prior to and early in pregnancy.

## Appendix

### Focus Group Guide

#### Introductions

Efficacy vs. effectiveness – reference to PowerPoint presentation.

1. Are you currently involved in delivering any of the interventions presented?
  - Can you tell me about them?
2. How effectively do you think they are delivered?
  - Coverage *–* are the right patients being contacted at the right time? *Service reach/accessibility and patient/client attendance.*
  - Provider compliance – is practice consistent across the profession and in line with the evidence base?
    *High quality patient/client and provider interaction.*
  - Patient compliance - do patients adhere to advice and interventions?
3. In relation to your role, which one or two of these interventions should be the focus of improvement?
  - What are the barriers to effective implementation?
  - What are the actions required?
4. Is there anything else you would like to add, for example, tell me about a time when something worked well re: preconception care?
